# Valorisation of Micro/Nanoencapsulated Bioactive Compounds from Plant Sources for Food Applications Towards Sustainability

**DOI:** 10.3390/foods12010032

**Published:** 2022-12-22

**Authors:** Valter F. R. Martins, Manuela E. Pintado, Rui M. S. C. Morais, Alcina M. M. B. Morais

**Affiliations:** CBQF—Centro de Biotecnologia e Química Fina—Laboratório Associado, Escola Superior de Biotecnologia, Universidade Católica Portuguesa, Rua Diogo Botelho, 1327, 4169-005 Porto, Portugal

**Keywords:** microencapsulation, nanoencapsulation, encapsulation techniques, bioactive compounds, plant, agro-industrial by-products, bioavailability, food applications, sustainability

## Abstract

The micro- and nanoencapsulation of bioactive compounds has resulted in a large improvement in the food, nutraceutical, pharmaceutical, and agriculture industries. These technologies serve, on one side, to protect, among others, vitamins, minerals, essential fatty acids, polyphenols, flavours, antimicrobials, colorants, and antioxidants, and, on the other hand, to control the release and assure the delivery of the bioactive compounds, targeting them to specific cells, tissues, or organs in the human body by improving their absorption/penetration through the gastrointestinal tract. The food industry has been applying nanotechnology in several ways to improve food texture, flavour, taste, nutrient bioavailability, and shelf life using nanostructures. The use of micro- and nanocapsules in food is an actual trend used mainly in the cereal, bakery, dairy, and beverage industries, as well as packaging and coating. The elaboration of bio capsules with high-value compounds from agro-industrial by-products is sustainable for the natural ecosystem and economically interesting from a circular economy perspective. This critical review presents the principal methodologies for performing micro- and nanoencapsulation, classifies them (top-down and/or bottom-up), and discusses the differences and advantages among them; the principal types of encapsulation systems; the natural plant sources, including agro-industrial by-products, of bioactive compounds with interest for the food industry to be encapsulated; the bioavailability of encapsulates; and the main techniques used to analyse micro- and nanocapsules. Research work on the use of encapsulated bioactive compounds, such as lycopene, hydroxytyrosol, and resveratrol, from agro-industrial by-products must be further reinforced, and it plays an important role, as it presents a high potential for the use of their antioxidant and/or antimicrobial activities in food applications and, therefore, in the food industry. The incorporation of these bioactive compounds in food is a challenge and must be evaluated, not only for their nutritional aspect, but also for the chemical safety of the ingredients. The potential use of these products is an available economical alternative towards a circular economy and, as a consequence, sustainability.

## 1. Introduction

In recent years, awareness has led to healthy options when buying food, and consumers, in general, prefer foods that do not only satisfy their primary needs, but that also include health effect promoters besides the nutrients, and that can replace synthetic additives by natural bioactive compounds, which may prevent illnesses. However, natural bioactive compounds, although safe, sometimes present an unpleasant taste or odour and instability. The direct incorporation of these compounds in food is often difficult, so nanoencapsulation constitutes an alternative to solve this issue. The use of nano- or microcapsules in food is a trend used in the cereal, bakery, dairy, and beverage industries, as well as packaging and coating [1]. Encapsulated compounds in foods include vitamins, essential fatty acids, minerals, flavours, antimicrobial agents, colorants, antioxidants, and polyphenols [2]. The incorporation of nutraceuticals [3] in food presents an issue, as both the nutritional purpose and the chemical safety of ingredients must be considered and evaluated [1].

The nanoencapsulation of bioactive compounds has brought large improvements to the food, nutraceutical, pharmaceutical, and agriculture industries. This technology allows the protection of bioactive compounds applied to different matrices and the control of their release. The elaboration of bio capsules with natural bioactive compounds is a challenge nowadays. The use of bioactive compounds extracted from by-products is economically interesting in the scope of a circular economy approach, and is more sustainable for the natural ecosystem [4,5].

Nanoencapsulation serves at the same time to target cells, tissues, or organs and to control the release and delivery of bioactive molecules. It also serves to protect the bioactive compound from several reactions that can modify the molecule or alter its bioactivity. Factors such as temperature, acidity, hydrolysis, and oxidation due to light exposure are some of the hazards for bioactivity. The advantages of this technology include (i) allowing targeting all the bioactive beneficial characteristics of the compounds to specific targets, (ii) controlling the release of the bioactive components, (iii) allowing the concentration of the compound to be decreased, thus preserving it in hostile mediums and maintaining its natural characteristics, (iv) preventing interactions between the bioactive molecules and other food ingredients, and (v) presenting a high solubility and dispersibility [1].

Nanoencapsulation deals with several types of materials on the atomic, molecular, and supramolecular scale, normally from 1 to 100 nm; nevertheless, some nanocapsules may present sizes up to 600 nm [6]. Nano refers to the size of 10^−9^ m, and the British standardization body defines nanotechnology as the conceptualisation, elaboration, characterization, and application of the products (nanocapsules) and systems [7].

In the last years, the food industry has applied nanotechnology in diverse forms to improve food texture, flavour, taste, consistency, nutrient bioavailability, and shelf life using nanostructures in stable emulsions. In addition, nanotechnology also presents well-known benefits in the fields of nutraceuticals, food microbiology, and nutrient delivery, by improving nutrient absorption in the gastrointestinal tract [8]. In food packaging, nanoencapsulation has improved materials by enhancing their mechanical strength and by producing antimicrobial films and nanosensors to detect pathogens or specific compounds, e.g., indicators of disease conditions, which are important tools to enlighten consumers about food safety issues [9]. 

Actually, not only value-added components of foods are in focus. It is also possible to remove undesirable components from modified functional foods, such as sugars, salt, and fats to obtain more healthy products.

It is important to distinguish microencapsulation from nanoencapsulation, with the principal difference being the size, but with the two also having different functions. The dimensions that distinguish microparticles from nanoparticles is still under debate. In microencapsulation, the scale varies between 1 and 8000 µm (Table 1). Other authors, such as Rossi et al. [5], have stated that nanocapsules and microcapsules are 10–1000 nm and 3–800 µm in diameter, respectively. Shishir et al. [10] have claimed that microcapsules stay in the range of 1 to 1000 µm, sub-microcapsules range from several hundred nanometres to less than 1 µm, and nanocapsules range from one to several hundred nanometres. Some authors have proposed a diameter inferior or equal to 100 nm for nanoparticles [11] and the range for colloid associations as 5 to 100 nm to be considered a nanoparticle [12]. The functionalities of microencapsulation are (i) the protection of bioactive compounds; (ii) the control of the release profile; (iii) the masking of undesirable flavours; (iv) the improvement of flow properties; (v) an increase in the shelf life; and (vi) product enrichment with specific nutrients. Very similarly, the functions of nanoencapsulation are (i) to reduce the practical size and create a restricted distribution of particles; (ii) to increase the surface area; (iii) to improve the delivery of bioactive molecules; (iv) to increase the bioavailability of encapsulated compounds; (v) to increase the physical stability and extend the shelf life; (vi) to increase precision targeting and to promote passage through fenestrated cells; (vii) to strengthen the barrier between bioactive and other compounds; and (viii) to improve intracellular absorption [10].

This review aims to perform a critical analysis of (i) the different methodologies to carry out micro- and nanoencapsulation for food applications, classifying these methodologies according to top-down (TD) and/or bottom-up (BU); (ii) the principal types of encapsulation systems; (iii) the natural plant sources of bioactive compounds of interest for the food industry to be encapsulated, enhancing the agro-industrial by-products; (iv) the bioavailability of the micro- and nanocapsules, presenting the various mechanisms for the release of the bioactive compound; and (v) the main techniques used to analyse these micro- and nanocapsules, and their respective advantages.

## 2. Micro- and Nanoencapsulation Methodologies

Nanoencapsulation is a process by which an active agent is coated by carrier material in order to form capsules on the nanometric scale. The coated materials are also designated as the core, fill, or internal phase, while the carrier materials are known as the wall material, membrane, capsule, shell, matrix, or external phase [10].

In nanoencapsulation, two aspects are normally relevant; one is the selection of the encapsulating material and the other is the specific encapsulation technique. For food applications, the encapsulating materials must be “generally recognized as safe” (GRAS). Nevertheless, it is of relevance as well to consider the encapsulated compound concentration, the stability of the capsule, its function in the food application, the target aimed for, and the efficiency of delivery [10]. The nanocapsule can be formed by biopolymers, such as proteins, carbohydrates, fats, and other organic and inorganic materials [1]. Emulsification, coacervation, inclusion complexation, emulsification solvent evaporation, extraction, nano-precipitation, electro-spraying (spray drying), and electro-spinning are the most common encapsulation techniques used for bioactive compounds [8]. The chosen technique varies according to the type of bioactive compound that is encapsulated, for example, whether it is volatile or not, the size of the nano- or microcapsule, the type of shell material, and other parameters. Nanoencapsulation is an area in constant innovation, and many recent studies have proposed new formulations or new encapsulation systems for the preservation of bioactive compounds with improved functionalities [10].

There is no standard technique to perform nanoencapsulation, and to choose a method, the type and characteristics of the active compound, which will be the core of the particle, must be taken into account; regarding the carrier material, its molecular weight, polarity, solubility, structure, and encapsulation efficiency need to be considered, and the particle size is also an important factor [5,10]. The principal techniques used for micro- and nanoencapsulation are listed in Table 1. Sometimes, two or more methods can be combined, such as emulsions used with spray drying or freeze drying.

**Figure 1:**
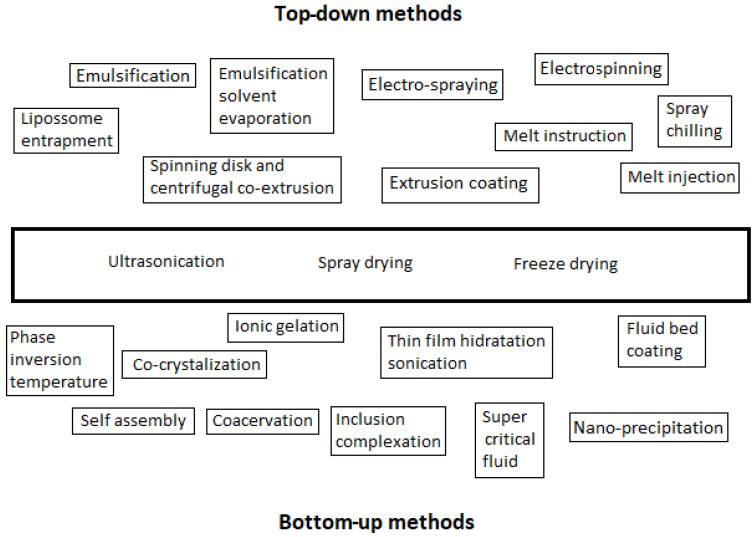
Techniques to produce micro- and nanocapsules, according to the top-down and bottom-up methodologies.

**Figure 2:**
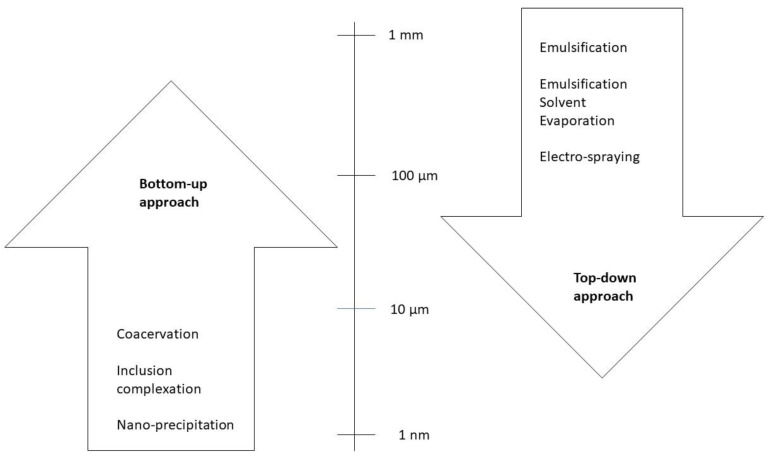
Size of the micro- and nanocapsules (adapted with permission from [39]. Copyright 2017, Elsevier).

Two approaches, the top-down and the bottom-up approaches, can be used to produce micro- and nanocapsules. Figure 1 presents several technologies used to produce micro- and nanocapsules according to these approaches. Figure 2 illustrates the difference between the two approaches relative to the size of the particles: the bottom-up one consists of the elaboration of capsules by the self-building or self-organization of molecules from small particles (nm) to big encapsulated aggregates (µm); the top-down approach consists of the opposite, involving the disintegration of bulk solids, liquids, or large particles (1 mm), in small particles (from 1 µm down to, for example, 200 to 450 nm of gelatine hydrogel particles [11]).

The top-down (TD) approach goes from a large-structure material to a small-structured one (Figure 2) through a size reduction and shaping of the structure through mechanical ways, such as milling, shredding, and grinding. It may use three types of forces to disrupt the particles: shear, impact, and compression. TD methodologies include emulsification, solvent evaporation, and extrusion. The latter, for example, creates small biopolymer particles in a solution by forcing it to pass through a nozzle into a gelling environment, with the size of the particles depending on the diameter of the needle, the flow rate, and the viscosity of the solution. Ultrasounds can be used to break up the polymer solution stream. Another process used is homogenization, which involves forming an emulsion by joining two immiscible liquids. This emulsion can be gelled by temperature management or by adding gelling agents, with the possibility of being an oil-in-water emulsion (normally, for proteins or polysaccharides) or a water-in-oil emulsion (hydrogels of 200–450 nm), but it can also be a water-in-water-in-oil or a water-in-oil-in-water emulsion [11]. Hydrophilic or hydrophobic compounds can be encapsulated by this TD methodology. However, it requires special tools and exerts low control over the particle size and structure. It may be applied only for some types of matrices. Furthermore, grinding and milling are not recommended for high-value and/or sensitive bioactive compounds because of the pressure and mechanical stress, which may induce damage [10,11].

The bottom-up (BU) methodology consists of the association of small particles (Figure 2) through self-building and self-organization. These can be affected by pH, temperature, the concentration of the encapsulated compound, and the ionic strengths. This approach includes spray drying, supercritical fluid expansion, inclusion complexation, coacervation, and nano-precipitation. These techniques consume less energy than top-down methods and allow control over the particle size, distribution, and structural morphology [10,11]. Coacervation is the process by which two oppositely charged biopolymers interact through electrostatic attraction, but hydrophobic interactions and hydrogen bonds may also occur [11]. Inclusion complexation is the molecular encapsulation of a bioactive molecule in the cavity of a host molecule. Drying is another technique, and it can be spray drying or freeze drying. Spray drying consists of atomizing a solution of biopolymers and bioactive compounds into fine droplets, forming microspheres or microgels with a size of 1 to 10 µm. Freeze drying includes freezing, sublimation, and desorption, requiring capsules with a high porosity, which affects the stability of the enclosed bioactive component and its retention efficiency. In a fluid gel formation, which includes thermal and ionic gelation, capsules are formed by applying shear forces on a biopolymer solution [11].

From a critical analysis of the various techniques to carry out micro- and nanoencapsulation (Table 1), it is possible to conclude that most methodologies begin by producing an emulsion through the junction of immiscible liquids or the solubilisation of solids, and then a specific technique is applied. For example, techniques such as nanoemulsion, ultrasonication, and freeze drying [40]; ionic gelation, ultrasonication, and freeze drying [41]; or ionic gelation and freeze drying [42] may be simultaneously used.

The literature underline that some techniques are used in both top-down and bottom-up methodologies, such as ultrasonication, spray drying, and freeze drying. However, there are others, such as ionic gelation and phase temperature inversion (some authors call it “fluid gel formation”, e.g., Joye et al. [11]), for which the classification as a TD or BU approach is non-consensual. Based on the principle of the nature, which consists of a powder solubilizing in a solvent, and taking into consideration that these methods use temperature inversion or ionic strength to form a gelling substance, they were classified as BU methodology in the present review work. 

The TD methodology involves the use of precise tools and specified equipment, which allow size reduction and structure shaping. However, it is a more expensive technique due to the costs of the equipment and its maintenance. Another issue is the elaboration of particles with a well-defined structure. Furthermore, grinding and milling may not be suitable for sensitive bioactive ingredients. While using a BU approach, the aggregates are built by the self-organization of the molecules, and this is influenced by several factors. This approach allows the production of very fine particles, thus controlling the size, morphology, and physical state. In addition, the risk of contamination is significantly reduced compared with the TD approach, and BU methods require higher energy than TD ones [11].

Another important issue is how to choose the technique to use, and here it may be suggested that the final function of the encapsulated product, such as gelling or in the form of a powder or a solution for spraying or dipping the food product, is most likely what dictates this selection.

Either technique of micro/nanocapsulation must allow a high loading capacity, a high encapsulation efficiency, and the stability of the encapsulated system, while providing a long shelf life, biocompatibility, and the desired release characteristics of the encapsulated active compound [10].

## 3. Nanoencapsulation Systems

Encapsulation can be affected by the size, shape, and internal structure of the capsules, their physicochemical stability, and their entrapment and release behaviours, with their biological activity being important as well. It is possible to associate the nanocapsule to the methodology that is used to make the aggregates. 

**Figure 3:**
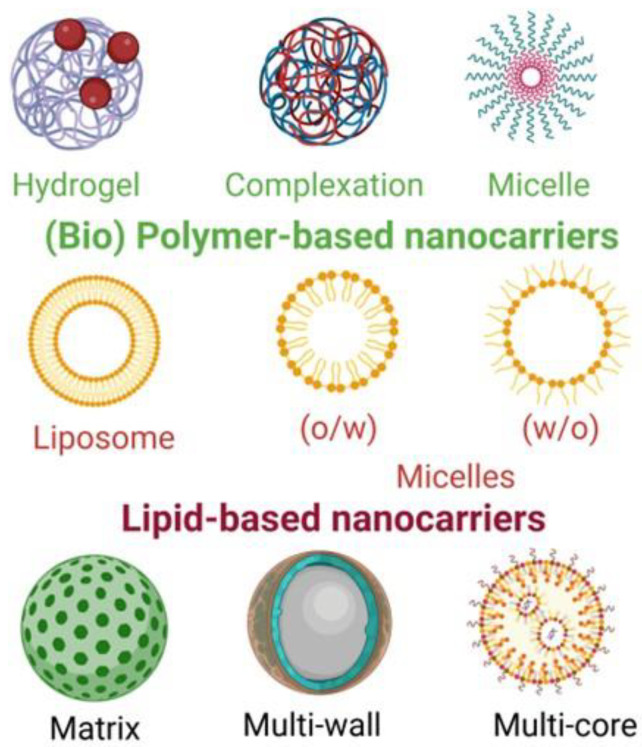
Some important nanocarriers for bioactive compounds used in food applications (created with BioRender.com, accessed on 4 December 2022).

**Figure 4:**
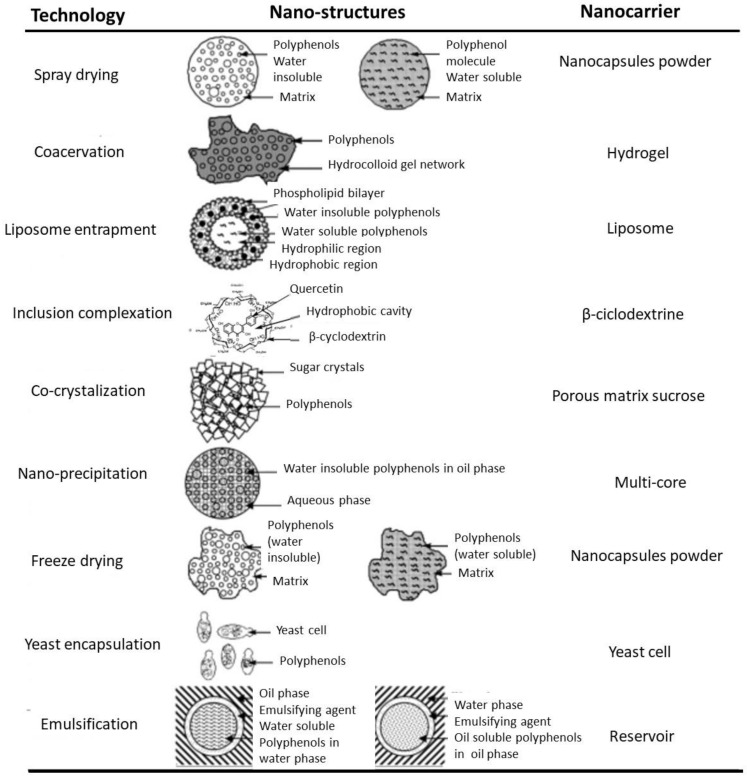
Nanostructures and nanocarriers for some nanoencapsulated bioactive compounds (adapted with permission from [25]. Copyright 2010, Elsevier).

Nowadays, several techniques are available for producing nanocapsules, as well as several materials to use as the shell of these aggregates, allowing various types of nanocapsules. The principal types are mentioned in the following text and are illustrated in Figure 3 and Figure 4. The characteristics of the compound to be encapsulated and the objective for its use condition the choice of the encapsulation system, e.g., the polarity of the compound and its hydrophilicity or lipophilicity. There is a high variety of carriers in nanoencapsulation, natural compounds or synthetic polymers, with principal characteristics in the text below and illustrated in the next figures.

There are several types of nanoencapsulation systems:

*Reservoir and matrix* (Figure 3): the active compound, surrounded by a polymeric membrane, is in a single hollow chamber in the reservoir system, which is called the capsule, single core, mono core, or core shell. The active compound is distributed in the encapsulated material, but it can also exist on the surface of the matrix system, called the sphere or particle. Both the reservoir and matrix may be combined, resulting in a multilayer form or coated matrix [5,10,43].

Emulsion (Figure 4): there are two immiscible phases in an emulsion: the disperse phase and the continuous one. The disperse phase droplets can entrap the bioactive compound and the continuous phase protects the loaded droplets from the environment. Two types of this emulsion are the water-in-oil and oil-in-water types. The disadvantages of these are their thermodynamic instability and susceptibility to destabilization. Multilayer emulsions, multiple emulsions, and nano emulsions can be formed [5,10,44].

Lipid nano-particles (Figure 3): in preparation, this system is similar to the emulsion systems, with lipophilic active compounds being spread in a mixture of solid and liquid forms of lipids with emulsifiers. Nanoparticles can be composed of solid lipids and carriers can be nanostructured lipids. To produce these particles, high-energy consumption may be used, such as high-pressure homogenization, micro-fluidization, or sonication methods; low-energy consumption methods, such as the phase inversion temperature, microemulsion, solvent diffusion or injection, and supercritical fluid technology, may also be used [10,44].

Lipid vesicular carriers (Figure 3 and Figure 4): among the diverse vesicular carriers, such as niosomes, bilosomes, transferosomes, ethosomes, and phytosomes, liposomes are the most used in food. Liposomes contain a hydrophilic head (polar) and a hydrophobic tail (non-polar), acting as a semi-permeable membrane, which separates the inner aqueous phase from the external water phase. The methods used to produce liposomes are high-pressure homogenization, micro-fluidization, electro-spraying, supercritical carbon dioxide technology, and ethanol injection [10,44].

Hydrogel particles (Figure 4): this system is a three-dimensional and cross-linked polymeric network that is able to quickly absorb water and to hold it in less favourable conditions (heat, pressure). Natural polymers have attracted more interest than synthetic ones. Polysaccharide-based and protein-based polymers are the most used to prepare hydrogel particles through gelation. These techniques allow the encapsulation of both hydrophilic and hydrophobic bioactive compounds [10,44].

Biopolymer-based systems (Figure 3): this system consists of polyelectrolyte complexes formed by electrostatic interactions among oppositely charged polymers, such as carrageenan and protamine, solubilizing the nutraceutical compounds in either a positively or negatively charged biopolymer. Biopolymer substances, such as amylose, starch, pectin carrageenan, and chitosan, can be used. 

Protein carbohydrates: protein carbohydrates are self-built structures composed of anionic polysaccharide and cationic protein surface groups, which may be produced by thermal denaturation or aggregation [44].

Molecular inclusion complexes (Figure 3 and Figure 4): molecular inclusion complexes are other encapsulated forms that are less used, such as cyclodextrins, but there are also nanofibres, nanotubes, and micelles [10].

The stability of a nanoencapsulation system may be inferred through the potential zeta, which is a physical property of particles in colloidal dispersions, influenced by the nanocapsule composition and the medium surrounding them [45].

## 4. Micro- and Nanocapsules from Natural Products

The use of “generally recognized as safe” (GRAS) materials is important for producing safe nanocapsules for food applications, along with the nutritional quality and stability. Polysaccharide-based carrier agents are natural carbohydrate polymers composed of various monosaccharides with glycosidic bonds and can be found in plants, animals, algae, and microorganisms. Some examples are starches from plants and the dextrins, maltodextrins, and cyclodextrins used to encapsulate hydrophobic bioactive compounds. Another example is cellulose, but it presents the limitation of being poorly soluble in water. Other compounds are pectin, chitosan, alginate, and gums. Upadhyay et al. [46] studied *Cananga odorata* essential oil nanoencapsulated with chitosan, forming a nano-emulsion. They concluded that it inhibited the growth of *Apergilus flavus* and improved the antioxidant activity. These results demonstrated that this formulation improved the shelf life of stored food against *Apergilus flavus*, which produces aflatoxin B1, and lipid peroxidation, turning natural nanocapsules into good candidates for edible coating formulations.

Protein-based carriers, which have a high nutritional value, may be GRAS materials. An example is whey protein, which has emulsifier proprieties; the caseins present in milk can constitute an excellent emulsifier. Good sources of edible proteins are gelatines, soy proteins, cereal (wheat, barley, and corn) proteins, potato protein, and pulse proteins (peas, chickpeas, lentils, beans, and lupins), and they present a high nutritional value. Another group of compounds with interest is the lipid-based carriers, fats, and oils. They may be polar lipids, such as monoglycerides and phospholipids, or nonpolar lipids, such as triacylglycerol and cholesterol. They are good emulsifiers that may be used in the encapsulation of bioactive compounds [10,45,47].

Natural resources are widely used as raw materials in industry. Nowadays, the reuse of natural bioactive compounds from by-products from the agro-food industry has increased, and under a circular bioeconomy perspective, these products are not wastes, but a source of economic-value compounds with a variety of applications. In some cases, by-products with low economic interest, but that are abundant, may also be used. The rational use of agro-industrial by-products in the nanobiotechnology field aims for the development of novel products and high-value-added processes. These agro-industrial by-products include fruit peels and seeds, pomaces, and straws, and they may be used as a source of high-value compounds, such as flavonoids, lycopene, and polyphenols. The main advantages of agro-industrial by-products are their abundance, low cost, biocompatibility, biodegradability, and, often, bioactivity. In this way, bioactive compound delivery systems may be obtained with relatively low production costs and environmentally sustainable management, due to the reuse of by-products [48].

Actually, around 1.3 billion tonnes of food are lost or wasted globally, costing USD 900 billion along the whole supply chain [49]. For instance, according to the World Bank, a 1% decrease in post-harvest losses might result in a USD 40 million reduction in this loss [50]. A more efficient use of agro-industrial by-products, considered residues, would most certainly contribute as well to reduce this loss, contributing at the same time to a circular economy by the conservation of the product value, materials, and resources in the economy for a long period with reduced waste generation, according to the Circular Economy Action Plan [49]. The extraction from these residues and the further micro- and nanoencapsulation of bioactive compounds with economic value for the food industry would represent a valorisation and a contribution to sustainable agro-food residue management.

### 4.1. Micro- and Nanoencapsulation for Functional Ingredient Delivery in Food Applications 

Wrong lifestyle choices often contribute to the development of obesity, type 2 diabetes, and cardiovascular diseases. An adequate diet through functional ingredients could manage prevention at an early stage and before a therapeutic intervention. However, some of these ingredients cannot permeate into the small intestine in a sufficient concentration for efficacy without an efficient oral delivery system. Nowadays, there are various solutions to respond to these problems, such as using low-permeable hydrophilic peptides and macromolecules (nanoparticles), or intestinal permeation enhancers, for these mucolytics appear as a solution and with potential application. Gleeson et al. [44] found that there is an opportunity for the nutraceutical industry to explore the progress of the pharmaceutical industry in drug delivery systems. Excipients or substances already tested in humans have a high potential to be used in formulations in a delivery approach. This might improve the solubility, stability, or permeability of those molecules. In diet, the bioavailability of bioactive compounds is a challenge, mainly for hydrophobic compounds. Nanoencapsulation presents itself as a solution for isolating a bioactive compound from its natural environment, e.g., a fruit or vegetable, and incorporating it into a suitable delivery system [51]. The technological use of edible nanocapsules might serve to preserve bioactive compounds that are adequate for food applications, protecting the antioxidant and antimicrobial characteristics of these compounds (Table 2).

From a critical analysis of the different micro- and nanoencapsulated bioactive compounds and related functionalities (Table 2), it is possible to conclude that the main purpose of using these compounds is to extend the shelf life of food products, due to their antimicrobial (mainly antifungal) and antioxidant activities. The principal technique of micro/nanoencapsulation is ionic gelation, probably because it is easy to apply in antimicrobial in vitro tests. Therefore, further studies are required on the application of encapsulates to the final food products. The studies reported in the literature are essentially in vitro studies on the effects of nano-aggregates on microorganisms; only a few studies apply to real food products. 

As already mentioned in this review, there are nanocapsules that carry natural compounds, mainly essential oils, but also extracts from plants and fruits, for food applications, as antimicrobials and antioxidants. However, there are only few studies on nanocapsules carrying natural compounds from agricultural by-products (Table 3). 

In addition, other types of compounds from by-products can be used in the shell composition of the nanocapsule. As mentioned above, soy, cereal, potato, and pulse proteins, polysaccharides, and lipid-based carriers are some examples of such compounds that may be used to encapsulate bioactive compounds. Table 3 shows some examples of shell materials, which can be obtained from agri-food by-products. 

It would be of the uttermost importance to carry out more research work on the extraction and preservation by micro- and nanoencapsulation of identified bioactive compounds from agro-industrial by-products. As previously mentioned, these agro-industrial by-products are actually under-used (e.g., as animal feed), often abundant, of low cost, and contain high-value compounds with antimicrobial and antioxidant properties, presenting a high potential for further re-use in the food industry. On the other hand, the economic features of agro-industrial by-product re-use have not yet been fully explored. Therefore, all these research studies would contribute to advances towards a circular economy and sustainability.

### 4.2. Nanoparticles with Natural Compounds from Agriculture By-Products

Searching for the use of by-products in nanotechnology brings along another issue that should be considered in this review: the use of nanoparticles from different sources, such as gold, silver, platinum, zinc, or iron, with these particles being coated with natural compounds from agricultural wastes [90,91]. The use of these by-products is usually related to antioxidant activity, while reinforcing the antimicrobial activity. Normally, the production of these nanoparticles is studied at variable conditions (temperature, pH) in order to optimize their antimicrobial and/or antioxidant activities [90,92]. 

The production of nanoparticles coated with natural bioactive compounds is very interesting and has been explored by several authors. For example, authors have investigated the use of silver antimicrobial nanoparticles in combination with grape seeds and pomace extracts [91,93], orange peel [91,92], persimmon seeds, peel and calyx extracts [94], *Persea americana* peel, *Beta vulgaris* peel and *Arachis hypogaea* shell [90], *Cocos nucifera* L. shell [95], sugar cane bagasse [96], banana peels extract [97], and mango peel extract [98].

Although most examples of nanoparticles are from inorganic materials, some may also be lipid-, protein-, or polysaccharide-based (see Section 3, nanoencapsulation systems, above). For example, Angourani et al. [99] used zein (corn protein) nanoparticles to entrap rosemary essential oil (entrapment efficiency ca. 71%) in order to prevent evaporation and preserve its bioactivity. Bilal et al. [100] suggested the use of starch, cellulose, and pectin as the main types of polysaccharide-based nanoparticles for food applications.

## 5. Micro- and Nanocapsule Bioavailability

Recently, research has focused on the development of new nanocarriers with potential applications in the formulation of functional foods. However, only a few products have been commercialized. Among all the nanocarriers studied, only five were approved by the Food Safety European Authority [101]; these included inorganic materials (iron, silver, calcium, magnesium, selenium, and titanium oxide) for nanoparticles, and organic nanomaterials (lipid-, protein-, and polysaccharide-based). This is because a major portion of the tests were in vitro and only a few were in vivo and, therefore, more of the latter studies were required to demonstrate the efficiency and safety of such nanoparticles/capsules [101].

The rule of five [101] outlines that 90% of the oral drugs in the pharmaceutical area must obey three out of four of the following guidelines, which allows compounds to be obtained that have a good oral bioavailability:

Molecular weight (MW) ≤ 500 Da;
Logarithm of its partition coefficient (log(P)) ≤ 5;
Hydrogen bond acceptors (HBA) ≤ 10;
Hydrogen bond donors (HBD) ≤ 5.

Some authors also add a polar surface area (PSA) ≤ 140 Å^2^ and a number of rotatable bonds (NRotB) ≤ 10. These guidelines were constructed based on the results of oral bioavailability [101,102].

The absorption of bioactive nanocapsules is conditioned by the properties of the bioactive compounds, the nanocapsule used, and the environmental conditions. Table 4 summarizes the principal steps of the absorption of nanoencapsulated compounds.

There are various mechanisms for the release of bioactive compounds from nanocapsules: diffusion, dissolution, erosion, swelling, osmosis, degradation, and fragmentation [103,105]: 

Diffusion: the diffusion of a bioactive compound from the interior to the exterior of an encapsulation system depends on the solubility in the encapsulated system and its permeability through the capsule material. The release rate of bioactive compounds can be affected by several factors, such as the bioactive compound characteristics (molecular weight, polarity, and vapour pressure); it depends also on the encapsulation system itself (polarity, physical state, interactions, and rheology).

Dissolution: two types of dissolution can be defined, with the first one being the encapsulation–dissolution-controlled system, in which bioactive compounds are encapsulated in dissolving materials and the dissolution rate is controlled by the solubility of the bioactive compounds and the physico-chemical proprieties of the carrier. In the second type, the matrix–dissolution-controlled system, the bioactive compounds are distributed uniformly through the particle, having an influence on the dissolution rate. 

Erosion: this release mechanism happens when the encapsulated system faces a specific environmental condition, the chemical degradation of the particle matrix, causing the release of the bioactive compound. It may be caused by various factors, such as physical (high temperatures), chemical (strong acids or bases), or enzymatic. There are two types of erosion: bulk erosion, where the degradation occurs throughout the entire particle, and surface erosion, where the degradation only occurs on the surface of the encapsulated compound. 

Swelling: the release of bioactive compounds happens when the capsule swells because of the solvent absorption; this release mechanism may be controlled by the selection of the polymeric matrix and the environmental conditions, such as temperature and pH. 

Osmosis: the release begins when an osmotic pressure is created by water absorption, triggering the release of bioactive compounds. Ultimately, this type of release mechanism is comparable with a solvent-activated release (swelling mechanism) because the particles absorb the solvent until rupture and then release the bioactive compounds. 

Degradation: degradation is the disruption of biomaterials by biological systems (microorganisms). The core compounds dispersed in the polymer matrix are released after the biodegradation of the polymer.

Fragmentation: the bioactive compounds are released from the encapsulation system when this is ruptured, due to environmental factors, such as pressure, pH, or enzymatic.

The release of nanoencapsulated bioactive compounds depends on the type of the bioactive compound and on the encapsulated system. The properties of bioactive compounds, such as the solubility, diffusivity, interior–exterior concentration gradient across the particle, interactions (repulsion forces between the bioactive compound and the encapsulation system), entrapment type of the bioactive compound inside the carrier, and size of the particle, will affect the release profile. The encapsulate properties can also condition the release of the bioactive compounds: the size, shape, structure, porosity, and composition can affect the release. Small nanoparticles tend to create an initial crack release followed by a slower one; however, big nanoparticles are degraded more slowly and display a slower diffusion of the bioactive compounds. The hydrophilicity or the hydrophobicity of a polymer may also cause strong interactions between the capsule and the bioactive compound, reducing the release rate. The oil phase of emulsion-based delivery systems controls the oil droplet polarity and increases the polymer molecular weight, with a high molecular weight displaying a reduced decomposition rate and causing an extended constant release of the bioactive compound over time. Some encapsulated bioactive compounds are soluble in either the oil or water phases and, as a consequence, they can leave the oil droplets when the emulsion is diluted in water. The environmental conditions, such as temperature, agitation, pH changes, and the presence of ions, can control the profile release of the bioactive compounds. For example, hydrogels are temperature-sensitive and pH-responsive. Other factors that can control the bioactive compound release are ultrasounds, light, oxidation, a reduction in the potential, and enzymes [103]. 

The effectiveness of nanocapsules requires evaluation, since they cannot be controlled with precision due to the complex processes of absorption, distribution, metabolism, and excretion [106]. 

## 6. Analysis of the Micro- and Nanocapsules

Nowadays, several techniques are available to analyse the structure of micro- and nanoencapsulated aggregates. Table 5 describes some of them, as well as their advantages in comparison to others, and what is possible to analyse by each technique.

## 7. Conclusions

The absorption of nutraceuticals in the human body increases with the use of micro- and nanocapsules and the protection of bioactive compounds, with a better control release being the major advantages of these systems. An attempt towards the optimization of the use of micro- and nanocapsules in food applications has been carried out over time. This review comprises a critical analysis of the different methodologies for performing micro- and nanoencapsulation for food applications, classifying them according to top-down (TD) and/or bottom-up (BU); the principal types of encapsulation systems; the natural plant sources, including agro-industrial by-products, of bioactive compounds of interest for the food industry to be encapsulated; the bioavailability of the micro- and nanocapsules; and the main techniques used to analyse them. It was possible to conclude that most methodologies of encapsulation begin by producing an emulsion, and then another technique is applied. Some techniques may be used in both TD and BU approaches, but others are not consensually classified. It is suggested that the final function of the encapsulated product is likely to dictate the selection of the micro/nanoencapsulation technique to be used. It was possible to conclude, as well, that among different food applications, the main focus of micro- and nanocapsuled bioactive compounds from plant sources, including agro-industrial by-products, is to extend the shelf life of food products, given their antimicrobial and antioxidant activities. Most studies have been performed *in vitro*, with the principal micro/nanoencapsulation technique applied being ionic gelation. Therefore, more studies on real food products and in vivo studies need to be carried out. In addition, research work on the use of encapsulated natural bioactive compounds obtained from agro-industrial by-products must be further reinforced, as it presents a high potential in food applications and in the food industry, and it may be an available economical alternative towards a circular economy, with sustainability for the natural ecosystem. Finally, the release of micro/nanoencapsulated bioactive compounds depends on several factors and the effectiveness of the nanocapsule requires evaluation, as it cannot be precisely controlled given the complex processes involved.

## Figures and Tables

**Table 1:** Micro- and nanoencapsulation methodologies.

Technology	Short Description	Shell and Core	Capsule Size	References
Electrospinning (TD)	Absence of heat; accumulation of nanoencapsulated electrospun fibre; may form a film.	Core: curcumin; Shell: zein	320 nm	[8,13]
		Core: vitamin E; Shell: chitosan	-	[14]
Electro-spraying (TD)	Electric field to distort the interface of the droplet; high direct voltage (0–30 kV); frequency, 30 kHz; discharge power, 45 kJ/m^2^.	Core: essential oils; Shell: cross-linking agent is calcium chloride	80 nm–900 µm	[15]
		-	5 nm–500 nm	[8]
		Core: quercetin; Shell: polyvinylpyrrolidone K10	5 nm–100 µm	[16]
		Core: vitamin E; Shell: chitosan	-	[14]
Emulsification (TD)	To dissolve bioactive and emulsifier in water or oil phase (w/o; o/w or o/w/o; w/o/w); nanoemulsions can be obtained using homogenizer, ultrasonicator, and microfluidizer.	Core: hemp cannabidiol; Shell: low-density lipoprotein (LDL) with carboxymethylcellulose (CMC)	200 nm–5000 µm	[8,17,18]
		Core: limonene; Shell: maltodextrin, starch, tween-40	50–1000 nm 543–1292 nm	[7]
Emulsification solvent evaporation (TD)	Emulsification of the polymer solution into an aqueous phase and evaporation of the solvent, causing the polymer precipitation as nanospheres.	Core: curcumin; Shell: poly lactide-co-glycolide (PLGA)	45 nm	[7]
		Core: quercetin; Shell: polycaprolactone (PLA)	130 nm	
Extrusion coating (TD)	To heat at 125 °C; to pour into a nitrogen-pressurized chamber; to extrude into a dehydrating liquid.	Core: citrus oils; Shell: corn syrup solids and glycerine	-	[19]
Liposome entrapment (TD)	To disperse lipid molecules in water, with bioactive agent in lipid or water phase; to reduce size by high shear or extrusion.	Core: vitamins, hormones, enzymes; Shell: one or more layers of lipids	-	[17,19]
		Core: vitamin C and iron; Shell: egg phosphatidylcholine, cholesterol, DL-α-tocopherol	10–1000 µm	[20]
Melt extrusion (TD)	To melt the coating; to disperse the bioactive in the coating; to incorporate the volatile compound in a thermoplastic matrix and force it through a nozzle.	Core: polyphenol; Shell: carbohydrate melt	-	[21]
		Core: essential oils, flavours; Shell: sucrose, corn syrup, gums, or maltodextrins	300–5000 µm	[22]
Melt injection (TD)	To melt the coating; to disperse the bioactive compound in the coating; to extrude through a filter; to cool or dehydrate.	Core: polyphenols; Shell: carbohydrate melt	-	[21]
		Core: essential oil; Shell: corn syrup	200–2000 µm	[22]
Spinning disk and centrifugal co-extrusion (TD)	Preparation of core and shell solutions; co-extrusion through nozzles.	Core: canola oil; Shell: alginate	150–8000 µm	[20,23]
Spray chilling (TD)	To dissolve the bioactive in a lipid solution at 32 to 42 °C; atomization (1.2 mm), 15 °C ± 2 °C, 5 bar pressurized air.	Core: vitamins and minerals; Shell: vegetable oils	-	[19]
		Core: bacteria (*Lactobacillus acidophilus* and *Bifidobacterium animalis* subsp. Lactis); Shell: vegetable fat	60.9–85.9 μm	[24]
Coacervation (BU)	To prepare oil-in-water emulsion with lipophilic bioactive in oil phase; to mix; to produce three immiscible phases; to cool; to cross-link.	Core: flavour oil; Shell: gelling protein	-	[8,17,19]
		Core: capsaicin; Shell: glutaraldehyde	100 nm	[7]
		Core: bovine serum albumin; Shell: chitosan and polyanion tripolyphosphate	200–580 nm	
Co-crystallization (BU)	Sucrose syrup (95–97° Brix); above 120 °C.	Coat: sucrose; Shell: polyphenols	Below 30 µm	[20,25]
Encapsulation by rapid expansion of supercritical fluid (BU)	To create a dispersion of bioactive and shell material in a supercritical fluid; 13 MPa, 62 °C, 1 h.	Core: caffeine; Shell: glyceryl monostearate	10–400 µm	[17,20,26]
		Core: lutein; Shell: hydroxyl propyl methyl cellulose phthalate	163–219 nm	[7]
Fluid-bed coating (BU)	Particles suspended at controlled temperature and humidity; high-speed air where the coating material is atomized; coalescence of drops to form a film.	Core: vitamins; Shell: gums, starches, or maltodextrins	50–500 μm	[19]
		-	20–200 μm	[17]
		-	100 nm to several mm	[27]
Inclusion complexation (BU)	In β-cyclodextrin, with hydrophobic centre and hydrophilic outer surface; water molecules replaced by less polar molecules; precipitate recovered and dried; binding by cyclodextrins, 200 °C.	Core: garlic and onion oils; Shell: cyclodextrin	-	[8,19]
		Core: docosahexaenoic acid, usnic acid; Shell: β-lactoglobulin and low methoxyl pectin, β-cyclodextrin	100 nm	[7]
Ionic gelation (BU)	Protonation of amino groups and interaction with negatively charged pentasodium triphosphate, establishing inter- and intra-molecular cross-linkages.	Core: *Zataria multiflora* essential oil; Shell: chitosan	Below 200 nm	[28]
		Core: jujube pulp and seed extracts; Shell: chitosan	130–270 nm	[29]
Nano-precipitation (BU)	Also called solvent displacement; precipitation of polymer from solution; only polymer-based wall material can be used.	Core: β-carotene; Shell: PLA, PLGA	80–100 nm	[7,8]
Phase inversion temperature (BU)	To use non-ionic surfactant that changes its solubility with temperature.	Core: *Origanum vulgare* essential oil; Shell: PEG-40 hydroxylated castor oil, polyoxyethylene 4-lauryl ether	20–200 nm	[30]
Self-assembly technique (BU)	Magnetically stirred slowly at room temperature overnight; heated at 90 °C for 20 min; dialyzed and lyophilized; 5 mg nanocapsule/mL to obtain the nanohydrogel.	Core: vitamin C; Shell: bovine serum albumin and citrus peel pectin	Below 100 nm	[31]
Thin-film hydration sonication (BU)	Lecithin and cholesterol in ethanol dried in vacuum; dried lipidic films hydrated with aqueous solution; probe sonication, 1 min.	Core: vitamin A palmitate; Shell: lecithin and cholesterol	76–115 nm	[32]
Freeze drying (TD and BU)	Oil-in-water emulsion stirred for 30 min, 500 rpm; sonicated, 24 kHz, for 120 s; dried.	Core: fish oil; Shell: whey protein and arabic gum	1.20–2.08 nm	[33]
Spray drying (TD and BU)	To disperse or dissolve the bioactive in aqueous coating solution; drying at 300–400 °C.	Core: flavours and edible oils, orange oil; Shell: arabic gum	-	[19]
		Core: lutein, catechin; Shell: maltodextrin, hydroxylpropyl methyl cellulose phthalate	80–219 nm	[7]
		Core: bacitracin; Shell: maltodextrin	3.1–6.6 μm	[34]
		Core: salbutamol sulphate; Shell: lactose	0.5–3 μm	
		Core: blackberry aqueous extract; Shell: arabic gum and polydextrose	-	[35]
Ultrasonication (TD and BU)	To use probe-type sonicator, 50 Hz, for 20 min.	Core: *Pelargonium graveolens* L. essential oil; Shell: chitosan	42.6 nm	[36]
Yeast encapsulation (TD and BU)	Storage of the emulsion-loaded yeast for the permeation (60 °C) of the flavours into the yeast; subsequent drying to seal the flavour-filled yeast.	Core: chlorogenic acid; Shell: yeast cell	5–10 μm	[37]
		Core: limonene; Shell: yeast cell	2 μm	[38]

TD—top-down approach; BU—bottom-up approach.

**Table 2:** Micro- and nanoencapsulated bioactive compounds, plant sources, and functionalities for food applications.

Bioactive Compound	Plant Source (Core)	Micro/Nanoencapsulation Method	Function	Reference
Cadinene, camphor, cinnamaldehyde, eugenol	*Cinnamomum zeylanicum* Blume essential oil EO	Oil-in-water emulsification; spray drying	Antimicrobial activity	[52,53]
Carnosic acid, carnosol, rosmadial, rosmaridiphenol, rosmarinic acid	*Rosmarinus officinalis* L. EO			[52,54]
Carvacrol, *γ*-terpinene, *p*-Cymene, thymol, *β*-caryophyllene	*Origanum vulgare* L. EO			[52,55]
Phenolics such as apigenin, quercetin	*Foeniculum vulgare* var. dulce (Mill.) EO			[52,56]
Menthol, menthone, menthofuran, menthyl acetate, piperitone	*Peppermint* EO; *Tragacanth* gum (shell)	Microemulsion and ultrasonication		[57]
Carvone, dillapiol, limonene	*Anethum graveolens* L. EO	Emulsion and freeze drying	Antimicrobial, anti-aflatoxigenic, antioxidant activities	[58]
Anethole, estragole, fenchone	*Pimpinella anisum* EO	Ionic gelation and ultrasonication	Antifungal activity	[59]
Carvacrol	*Thymus capitatus* EO	Nano-precipitation		[60]
Carvacol, cymene, thymol, *β*-fenchyl alcohol, *γ*-terpinene	*Origanum* *vulgare* EO	Phase inversion temperature		[30]
Carvacrol, *γ*-terpinene, thymol, *o*-cymene, *α*-pinene	*Zataria multiflora* EO	Ionic gelation		[28,61]
Elemicine, myristicine, thujano	*Myristica fragrans* Houtt. EO	Ionic gelation		[62]
Eugenol	*Eugenia caryophyllata* and *Ocimum sanctum* EO	Ionic gelation and ultrasonication		[63]
Linalool	*Homalomena aromatic* EO	Ionic gelation and freeze drying		[42]
Linalool, methyl chavicol	*Ocimum sanctum* EO	Emulsion and freeze drying		[64,65]
Menthol, menthone, menthofuran, pulegone	*Mentha piperita* EO	Self-assembly and ultrasonication		[66,67]
Cadinene, caryophyllene, cymene, limonene, phellandrene	*Schinus molle* L. EO	Ionic gelation	Antifungal, anti-aflatoxigenic activities	[68]
Camphor	*Ocimum canum* EO	Nano-emulsion, ultrasonication, and freeze drying		[40]
Eugenol	*Ocimum sanctum* EO			
Estragole	*Ocimum basilicum* EO			
Citral	*Cymbopogon citratus* EO	Ionic gelation, ultrasonication, and freeze drying		[41]
Geraniol	*Lippia rugosa* EO			
Terpineol	*Melaleuca alternifolia* EO			
Citronellyl formate, linalool, menthone	*Pelargonium graveolens* EO	Emulsion and ultrasonication		[36]
Cuminaldehyde, cymene, terpinene terpinen-7-al	*Bunium persicum* (Boiss.) B. Fedtsch EO	Ionic gelation		[69]
Linalool	*Cananga odorata* EO	Ionic gelation and freeze drying		[70]
Methyl cinnamate	*Zanthoxylum alatum* EO			
Thymol	*Thymus vulgaris* EO			
Anethole, estragole	*Illicium verum* EO	Ionic gelation	Anti-aflatoxigenic, antioxidant activities	[71]
Eugenol	*Clove* EO	Electrospray	Anti-mycotoxigenic (antifungal)	[72]
Benzyl acetate, linalool	*Conanga odorata* EO	Ionic gelation	Antifungal, antioxidant activities	[46]
Carvacrol	*Petroselinum crispum* EO	Nano-emulsion		[73]
Curcumin	*Curcuma longa* L. root	Spray drying	Antioxidant activity, colourant (fortified rice)	[74]
		Emulsion and ultrasonication	Food colourant	[75]

EO—essential oil.

**Table 3:** Micro- and nanoencapsulated bioactive compounds from agro-industrial by-products for the shelf-life extension of food products.

Bioactive Compound	Source/By-Product	Micro/Nanoencapsulation Method; Shell	Function	References
Coumarin, resveratrol, quercetin	Acerola/unused pulp; guava/peel; passion fruit/seeds	Emulsion evaporation solvent	Antimicrobial and antioxidant activities	[76]
Hesperidin, naringin, narirutin, neohesperidin	Orange/peel	Emulsion and freeze drying; Shell: maltodextrin, whey protein isolate		[77]
Anthocyanins	Mulberry/pomace	Extrusion; Shell: calcium alginate	Antioxidant activity	[78]
Anthocyanins, ascorbic acid, carotenoids, flavanols, flavonols	Cherry/pomace	Ultrasonication; Shell: maltodextrin		[79]
Anthocyanins, ascorbic acid, carotenoids, flavanols, flavonols	Sour cherry/pomace	Ultrasonication and freeze drying; Shell: maltodextrin or arabic gum		[79]
Ascorbic acid, carbohydrates, phenolics, riboflavin, thiamine	Jujube/pulp and seed extracts	Ionic gelation; Shell: chitosan		[29]
Ascorbic acid, flavonoids (eriocitrin hesperidin, naringin, narirutin), phenolics, pectin	Orange and lemon/peel	Emulsion; Shell: maltodextrin or arabic gum		[80]
Cyanidin, delphinidin, malvidin, pelargonidin, peonidin, petunidin	Black carrot/pomace	Emulsion, ultrasonication		[81]
Flavonoids, phenolics	Fruit pomegranate/peel	Nanoemulsion; Shell: maltodextrin, whey protein isolate		[82]
Lycopene	Tomato/pomace	Spray drying; Shell: arabic gum or inulin		[83]
Monoterpene and sesquiterpene hydrocarbons (α-pinene, myrcene, α-humulene, (E)-caryophyllen)	Industrial hemp/essential oils (*Cannabis sativa* L.)	Nanoprecipitation; Shell: alfalfa protein isolate		[84]
Phenolics, flavonoids, flavones (nobiletin), polymethoxyflavones, (tangeretin, nobiletin)	Citrus (mandarin, lemon, lime, sweet orange)/pomace	Emulsion, ultrasonication; Shell: lipids (medium-chain triglycerides)		[85]
Phenolics (*p*-hydroxybenzoic acid, epicatechin gallic acid), anthocyanins	Blueberry/pomace	Emulsion; Shell: whey proteins		[86]
Polyphenols	Apple/pomace	Ultrasonication		[87]
Resveratrol	Grape wine/pomace	Spray drying; Shell: maltodextrin and milk proteins		[88]
Tyrosol	Olive/pomace	Emulsion and freeze drying		[89]

**Table 4:** Nanocapsule absorption in the human body ([103,104]).

Body Target	Example of Nanocapsule	Nanocapsule Degradation for Release of Bioactive Compounds	Goal
Mouth	Hydrogel, biopolymeric particles	Mechanical disruption, pH and temperature changes, enzymatic degradation (1 to 5 min)	Release of flavour
Stomach	Molecular complexes: protein-based hydrogel particles, protein-based biopolymeric particles	pH decrease, ionic strength changes, mechanical disruption, enzymatic degradation (30 min to 4 h)	Release of acid-stable nutrients
Small intestine	Hydrogel and biopolymeric particles produced with digestible proteins and polysaccharides	pH increase, ionic strength changes, enzymatic degradation (1 to 2 h)	Release and protection of acid-sensitive nutrients
Colon	Dietary fibre-based, hydrogel particles, biopolymeric particles	pH increase, enzymatic degradation, bacterial degradation (12 to 24 h)	Delivery of probiotics

**Table 5:** Techniques to analyse the nanocapsules.

Encapsulated Bioactive Compound	Technique of Analysis	Advantages	Objectives	Reference
Volatile and non-volatile bioactive compounds of food	Spectroscopy UV/Vis, GC, GC-MS, LC, LC-MS, HPLC	High reproducibility; versatile approach to performing quantitative analyses	Size, morphology, loading capacity, entrapment efficiency, stability, structural composition	[107]
Liquid, solid, and semi-solid samples	Nuclear magnetic resonance (RMN)	Non-destructivity; non-invasiveness; high reproducibility; quantitative determination; no compound separation before analysis	Characterization of nanocapsules in different environments	[108,109]
Chitosan, folic acid, vitamin D3	Dynamic light scattering (DLS)	Easy preparation of samples; can measure particles with less than 1 nanometre	Particle size, particle size distribution, and relaxation in complex fluids	[110]
Lipid-based, protein/polysaccharide-based, nanofibers, nanotubes	Scanning electron microscopy (SEM)	Scale of nanoparticles evaluated	Study of the morphology and surface structure	[111]
*Caranga odorata* essential oil encapsulated in chitosan; casein hydrolysate encapsulated in maltodextrin	High-resolution scanning electron microscopy (HR-SAM), Fourier-transform infrared spectroscopy (FTIR), X-ray diffraction (XRD)	High sensitivity and reproducibility; HR-SAM allows the matrix-type structure of particles to be seen	Characterization of the nanocapsules; identification of the functional groups and covalent interactions; study of the surface morphology	[46,112]
Encapsulated systems	Atomic force microscopy (AFM)	Very high scanning resolution; topological image and nano-chemical analysis	Study of the surface and structure of the nanocapsule	[113]
	X-ray photoelectron spectroscopy (XPS)	High sensitivity and accuracy	Surface characterization	[114]
	Confocal laser scanning microscopy	Non-destructivity	Loading capacity, observation of three-dimensional internal structures	[115]

GC—gas chromatography, LC—liquid chromatography, MS—mass spectrometry, HPLC—high-performance liquid chromatography.
